# Thermal ablation with configurable shapes: a comprehensive, automated model for bespoke tumor treatment

**DOI:** 10.1186/s41747-023-00381-6

**Published:** 2023-11-07

**Authors:** Iwan Paolucci, Milica Bulatović, Stefan Weber, Pascale Tinguely

**Affiliations:** 1 ARTORG Center for Biomedical Engineering Research, University of Bern, Bern, Switzerland; 2 Department of Visceral Surgery and Medicine, Inselspital, University Hospital Bern, Bern, Switzerland

**Keywords:** Hyperthermia (induced), Liver neoplasms, Microwaves, Radiology (interventional), Robotics

## Abstract

**Background:**

Malignant tumors routinely present with irregular shapes and complex configurations. The lack of customization to individual tumor shapes and standardization of procedures limits the success and application of thermal ablation.

**Methods:**

We introduced an automated treatment model consisting of (i) trajectory and ablation profile planning, (ii) ablation probe insertion, (iii) dynamic energy delivery (including robotically driven control of the energy source power and location over time, according to a treatment plan bespoke to the tumor shape), and (iv) quantitative ablation margin verification. We used a microwave ablation system and a liver phantom (acrylamide polymer with a thermochromic ink) to mimic coagulation and measure the ablation volume. We estimated the ablation width as a function of power and velocity following a probabilistic model. Four representative shapes of liver tumors < 5 cm were selected from two publicly available databases. The ablated specimens were cut along the ablation probe axis and photographed. The shape of the ablated volume was extracted using a color-based segmentation method.

**Results:**

The uncertainty (standard deviation) of the ablation width increased with increasing power by ± 0.03 mm (95% credible interval [0.02, 0.043]) per watt increase in power and by ± 0.85 mm (95% credible interval [0, 2.5]) per mm/s increase in velocity. Continuous ablation along a straight-line trajectory resulted in elongated rotationally symmetric ablation shapes. Simultaneous regulation of the power and/or translation velocity allowed to modulate the ablation width at specific locations.

**Conclusions:**

This study offers the proof-of-principle of the dynamic energy delivery system using ablation shapes from clinical cases of malignant liver tumors.

**Relevance statement:**

The proposed automated treatment model could favor the customization and standardization of thermal ablation for complex tumor shapes.

**Key points:**

• Current thermal ablation systems are limited to ellipsoidal or spherical shapes.

• Dynamic energy delivery produces elongated rotationally symmetric ablation shapes with varying widths.

• For complex tumor shapes, multiple customized ablation shapes could be combined.

**Graphical Abstract:**

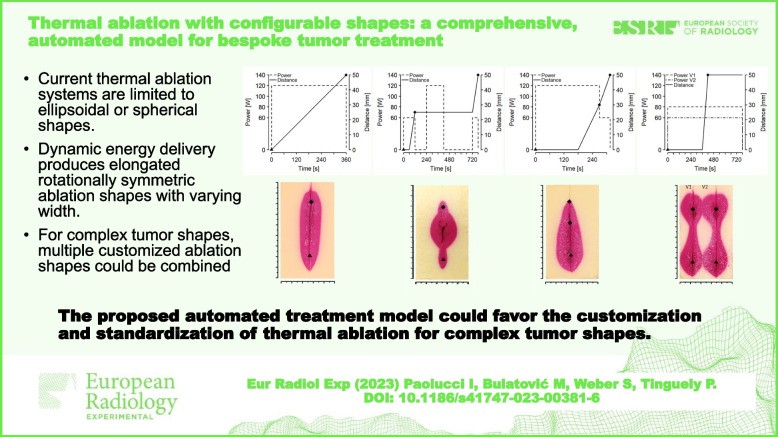

**Supplementary Information:**

The online version contains supplementary material available at 10.1186/s41747-023-00381-6.

## Background

Thermal ablation is an increasingly performed technique for the treatment of malignant tumors, primarily in solid organs such as the liver [1, 2]. Leading to similar oncological outcomes as the traditional approach of surgical resection for selected cases [3], it is now widely accepted also for “curative-intent” treatments of the most frequent liver tumors (hepatocellular carcinoma and liver metastases from colorectal cancer) and is included in the current clinical practice guidelines [4, 5]. The striking advantages of thermal ablation are its tissue-sparing nature, conserving healthy liver tissue and therefore allowing easy re-treatment in the frequent case of tumor recurrence [6]. It is also applicable with minimally invasive treatment accesses such as a percutaneous approach, further reducing treatment-related morbidity.

**Fig. 1:**
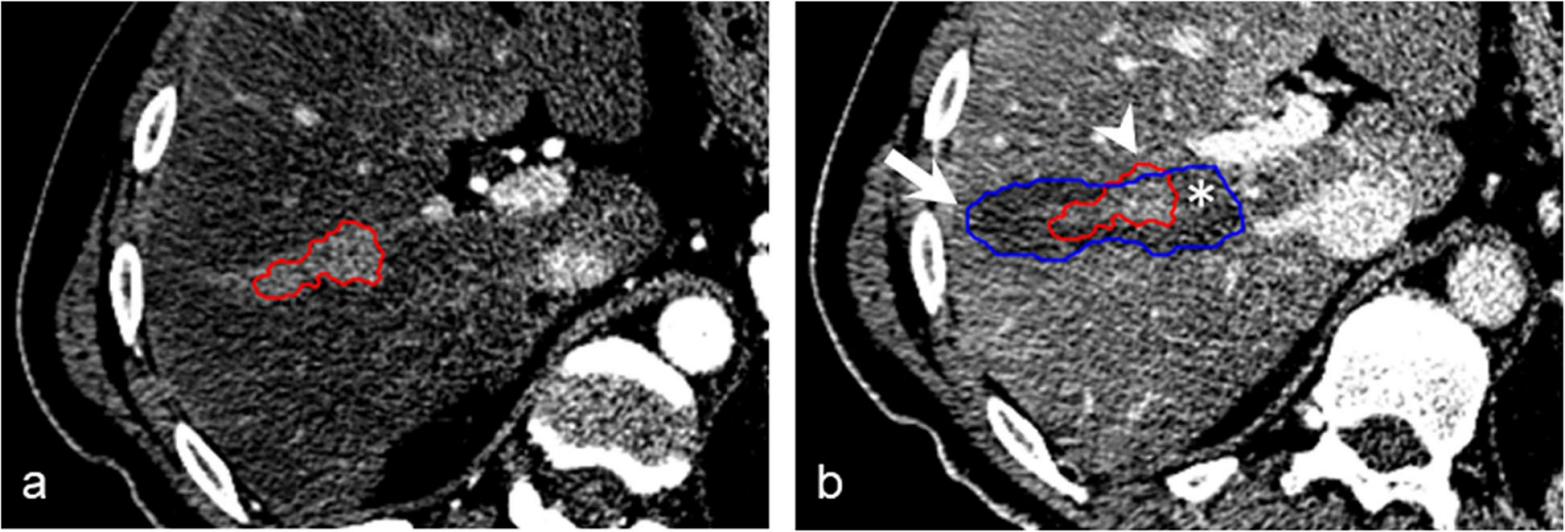
An irregularly shaped malignant liver tumor (red) was covered by two inadequate “standard” ellipsoid-shaped ablation volumes (blue), resulting in residual, untreated tumor tissue beyond the ablation volumes (arrowhead), unnecessarily ablated healthy tissue (arrow), and potential harm to critical structures (asterisk)

During thermal ablation, electromagnetic waves in the frequency range of radio waves (450–500 kHz) or microwaves (900 MHz, 2.5 GHz) are applied within and around the tumor via an ablation probe [7]. Interaction with the tissue converts electromagnetic energy into thermal energy, which above 60 °C causes coagulative necrosis of cancer cells [8]. The treatment aim is full coverage of the tumor by the ablation volume with an adequate ablation margin [9], to avoid incomplete ablation and local tumor recurrence. Meanwhile, thermal injury to surrounding critical structures, such as major blood vessels or bile ducts and necrosis of healthy liver tissue induced by the ablation energy, must be avoided (Fig. 1).

Finding this balance requires a high degree of treatment precision, representing the main challenge and reason why many clinicians still have reservations toward a broad application of thermal ablation for malignant liver tumors.

First, precision in tumor targeting. Many tumors are located in areas within the liver where conventional image guidance techniques for placement of ablation probes fail to reach accurately and safely, *e.g.*, below the diaphragm. In clinical reality, this often leads to tumors being labeled “non-reachable” and being precluded from thermal ablation therapy. The use of sophisticated stereotactic guidance technology for precise planning of ablation probe trajectories and accurate probe positioning with or without automated aiming devices [10] has addressed and solved this issue, leading to enhanced local tumor control when compared to traditional manual guidance techniques [10, 11].

Second, precision in the creation of adequate ablation volumes. With current thermal ablation devices, the size of the ablation volume can be increased by enhancing the applied energy; however, the radii of the typically ellipsoid ablation shapes are interlinked and cannot be modified independently. This leaves clinicians with a “nearest fit” solution, unsuitable for the mostly irregularly shaped tumors requiring ablation coverage around tumor “spikes” to avoid residual unablated tumors. Solving the problem with the creation of large ablation volumes and areas of necrosis can harm the surrounding structures and compromise safety [12, 13]. This “spatial challenge” of planning and creating adequate ablation volumes adapted to individual tumor shapes remains wholly unanswered on a technological level.

Third, precision in the evaluation of adequate ablation coverage. To allow immediate knowledge of the tumor coverage by the created ablation volumes and therefore treatment success, dedicated software algorithms applying advanced radiomics for quantitative ablation margin verification have been developed, which can be integrated into the before-mentioned sophisticated navigation solutions [9].

Aiming to create a solution for overarching precision from treatment planning to validation, we herein propose a comprehensive, automated treatment model for thermal ablation of malignant liver tumors. We present the development of an automated, dynamic energy delivery system aiming to create configurable ablation shapes bespoke to the individual tumor appearance, which currently represents the missing piece toward bespoke thermal ablation with a standardized approach. To this end, we consider the volumetric shape of the individual tumor and its anatomical surroundings as a guide to designing the clinically most effective treatment strategy. Proof-of-principle and *ex vivo* applicability of the system were investigated.

## Methods

**Fig. 2:**
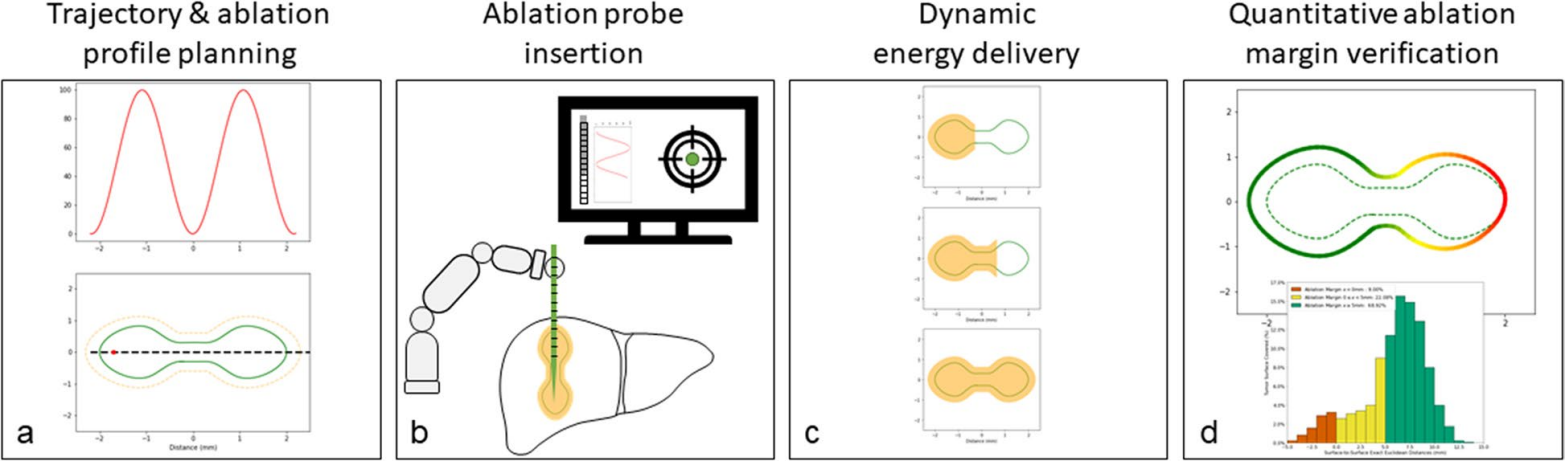
Four-step approach including (from left to right) (**a**) trajectory (black dashed line) and ablation profile planning (dashed orange line), (**b**) stereotactic ablation probe insertion, (**c**) dynamic energy delivery, and (**d**) quantitative ablation margin verification with identification of insufficiently ablated tumor

The proposed comprehensive treatment model for bespoke, automated thermal tumor ablation includes four separate steps, as illustrated in Fig. 2. Steps i, ii, and iv have in part been previously described and will be integrated into the automated treatment algorithm using a standard robotic system: (i) software supported trajectory planning [14], automated ablation profile planning (as proposed herein); (ii) automated ablation probe insertion [15]; (iii) dynamic energy delivery according to treatment plan (as proposed herein); and (iv) quantitative ablation margin verification [9, 16–18]. This will allow a fully standardized and automated algorithm from treatment planning to treatment verification for a given tumor.

### Dynamic energy delivery

#### System concept and design

The shape of each individual tumor and its anatomical surroundings are considered as a guide for effective planning of adequate ablation volumes, *i.e.*, for determining which cell volumes must be uniformly covered by thermal energy leading to temperatures above 60 °C and tumor necrosis. The thermal energy delivered during thermal ablation depends mainly on power, time, distance to the energy source (*i.e.*, the ablation probe), and tissue properties such as specific heat capacity or thermal conductivity [8].

**Fig. 3:**
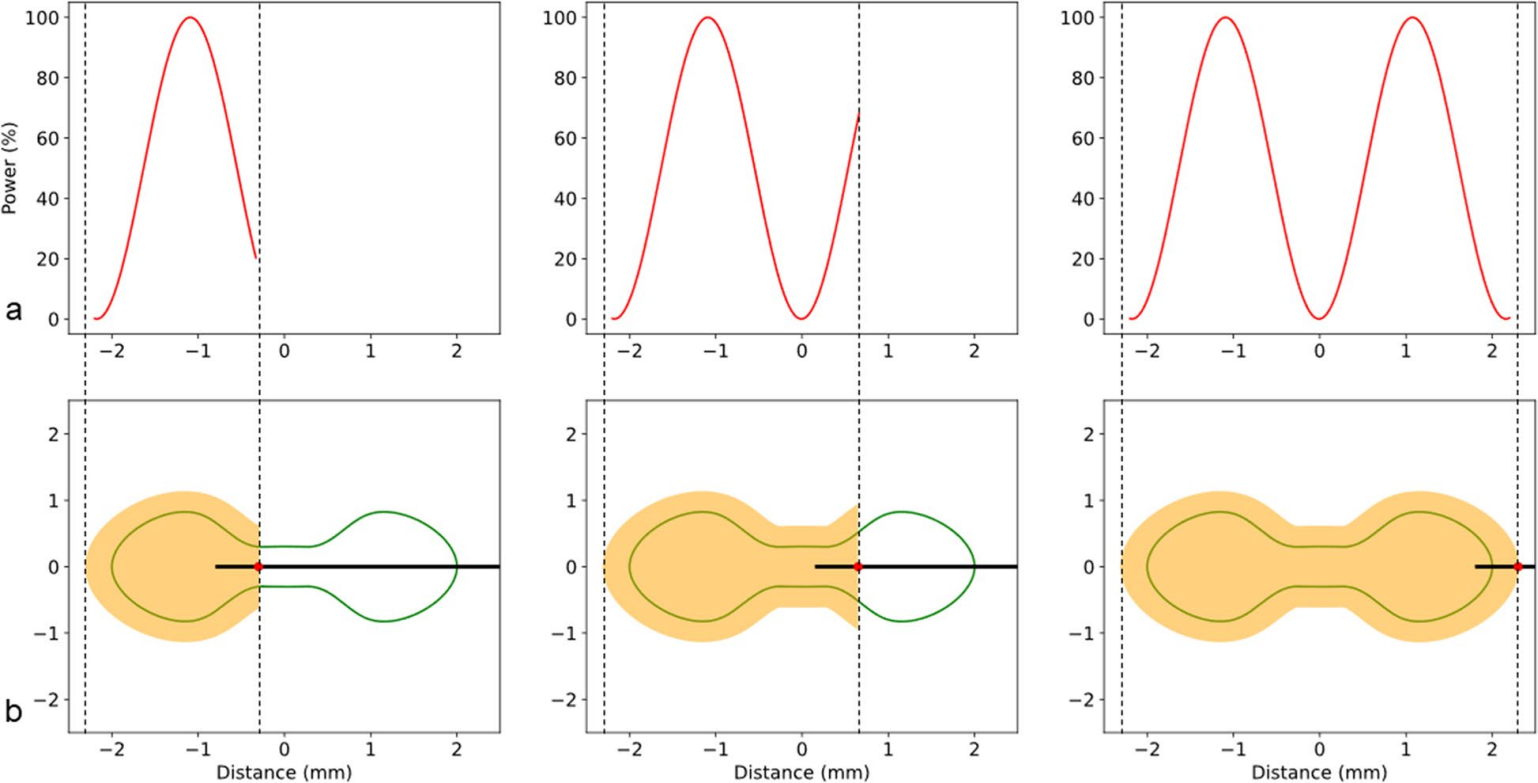
**a** A profile of the ablation power (red curve) varying depending on the location of the ablation probe. **b** Outline of a tumor (green) and a dynamically forming ablation shape (yellow) around the ablation energy source (red point) and along the longitudinal ablation probe trajectory (black line) moving from left to right

Based on these interactions, we propose a modification of the power and the location of the energy source over time, such that all cancer cell volumes receive adequate thermal energy according to a bespoke shape. The concept of the dynamic energy delivery system consists of creating a longitudinal ablation profile with the distance traveled by the ablation probe and the ablation power varying over time, as illustrated in Fig. 3. The ablation power and speed with which the ablation probe moves along a straight-line trajectory are modulated to compose an ablation volume following the shape of the targeted tumor. In that way, elongated volumes and wider shapes can be created in a longitudinal fashion to cover corresponding portions of the tumor. By superimposing such multiple volumes with varying longitudinal trajectories in a defined manner, any kind of complex ablation shapes can be created around critical structures or for more complicated tumor configurations.

**Fig. 4:**
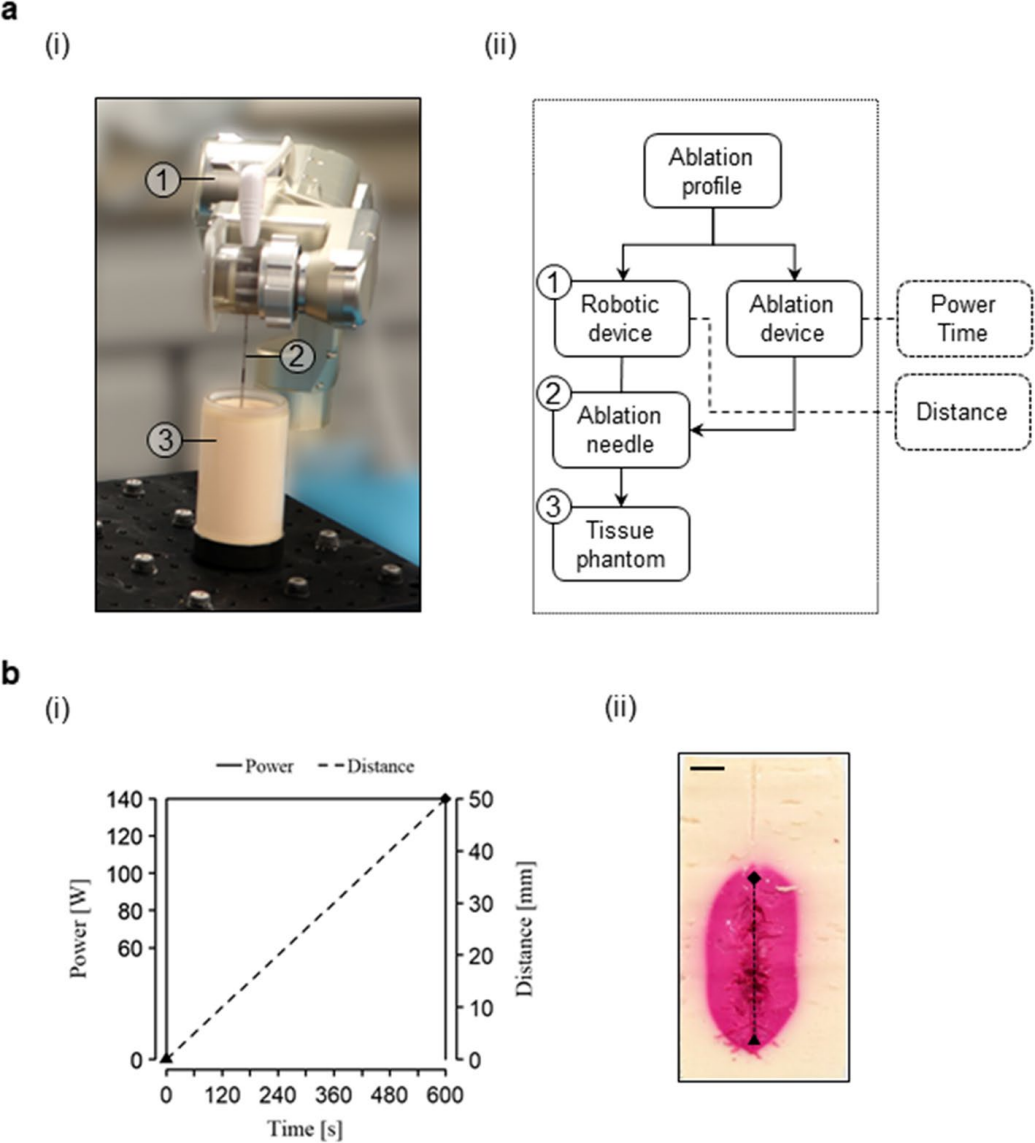
**a** Experimental setup (**a**,i) and workflow (**a**,ii). **b** Example of an ablation profile (**b**,i) and the resulting ablation volume (**b**,ii)

A standard robotic system with an integrated robotic arm can be used to align and move the ablation probe according to the defined ablation profile. A proposal of a potential system design with its components is shown in Fig. 4.

#### Fundamental principle

The ablation probe is displaced over time along a straight-line trajectory from the start point ${{\varvec{l}}}_{0}$ to the end point ${{\varvec{l}}}_{{\varvec{n}}}$. The location of the ablation probe at each point in time ${{\varvec{t}}}_{{\varvec{i}}}$ is denoted as ${{\varvec{l}}}_{{\varvec{i}}}$. The width of the ablation dependent on a specific location along the trajectory is then given by the following formula:

1 $${w}_{i}=\frac{1}{\sqrt{{{\beta }_{\mathrm{device}}\cdot \beta }_{\mathrm{tissue}}}}\cdot \sqrt{\frac{{P}_{i}}{{v}_{i}}}$$

where ${\beta }_{\mathrm{tissue}}$ is a coefficient depending on tissue properties, ${\beta }_{\mathrm{device}}$ is a coefficient of the device, $P$ is the output power of the device, and $t$ is the time when the probe passes through the point x on the trajectory. The coefficient ${\beta }_{\mathrm{tissue}}$ is derived from tissue properties and describes how much ablation width is produced per kJ of energy. The exact relationship between tissue (tumor, organ, *e.g.*, liver) and ablation energy expansion is a matter of ongoing research [15]. No single estimator derived from imaging or biopsy is known as of yet, leading to high variability in the expansion of ablation energy and resulting ablation volumes*.* Therefore, we propose a simplified model, which can be calibrated by experimental observations:

2 $$\mathrm{width}\left({p}_{i}\right)=\alpha + {\beta }_{\mathrm{power}} \cdot \mathrm{log}\left(\mathrm{power}\right)+{\beta }_{\mathrm{velocity}} \cdot \mathrm{log}\left(\mathrm{velocity}\right)$$

To calibrate an ablation device for a specific type of tissue the coefficients $\alpha , {\beta }_{\mathrm{power}}, {\beta }_{\mathrm{velocity}}$ must be estimated.

### Experimental setup

**Fig. 5:**
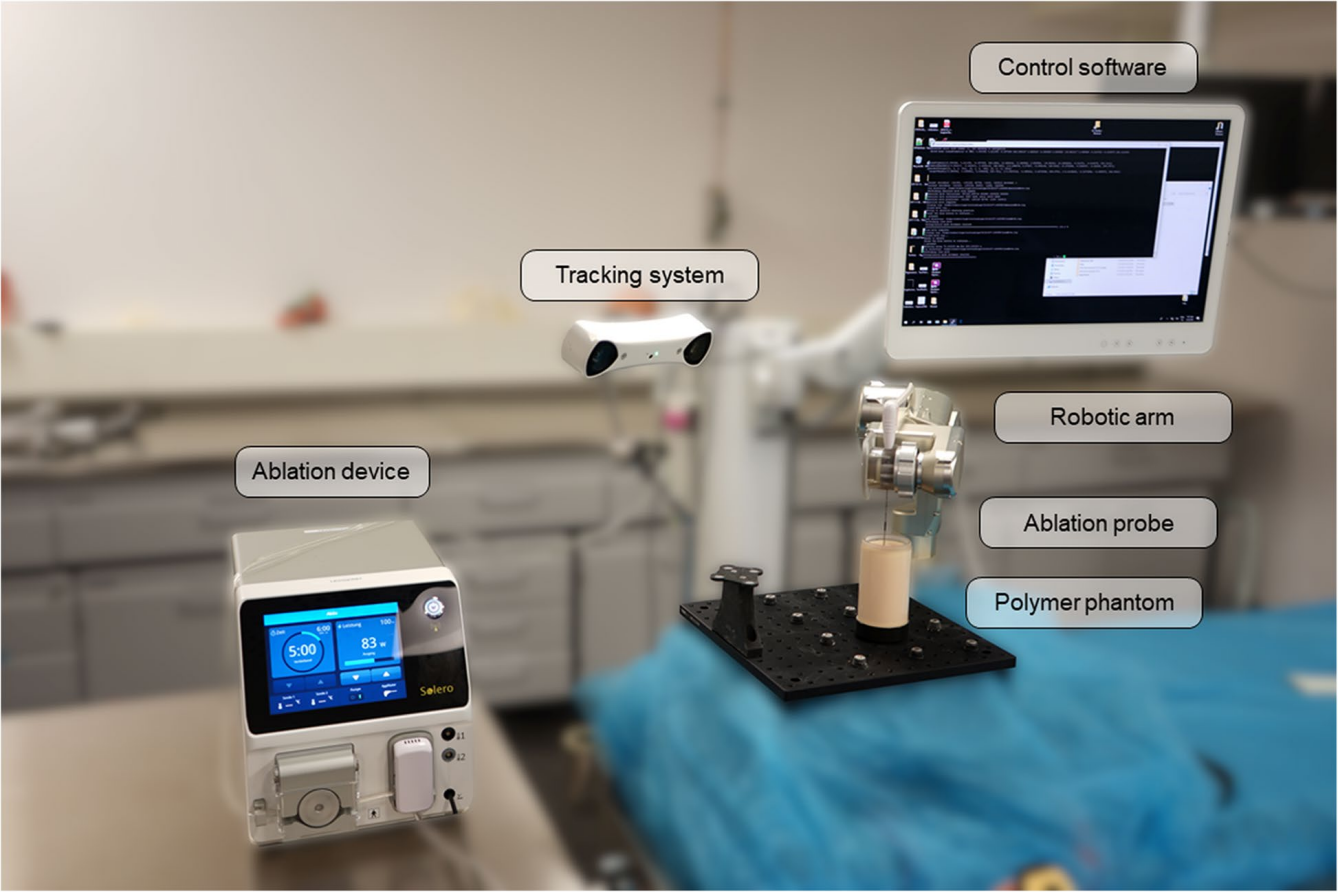
Potential setup to test configurable ablation shapes using a robotic device in a polymer phantom: microwave ablation generator (Solero, AngioDynamics, Latham, NY, USA) and corresponding ablation probe, 5 DoF robotic arm with a custom-made end effector to hold the ablation probe, optical tracking system (spryTrack 180, Atracsys, Puidoux, Switzerland), dedicated robot control software

A pilot experimental setup consisting of a thermal ablation system (Solero Microwave Tissue Ablation System, AngioDynamics, Latham, NY, USA), a surgical robot [19] and a tissue-mimicking phantom [20], was composed (Fig. 5). The ablation system comprised a microwave ablation generator and a water-cooled ablation needle, with two adjustable parameters: duration (maximum 6 min in one cycle) and power (60–140 W with 20 W increments). The power output displayed on the screen of the generator was monitored by a camera and recorded. The surgical robot used was a 5-axis lightweight (< 5 kg) robotic arm, which can be mounted on the side rails of a standard operating table or a computed tomography table, with robot control software available on a computer. The robot end effector was custom-built with a clamping mechanism to hold the ablation probe in place. Optical tracking, the most common tracking method for stereotactic liver ablation [10], was used to track the phantom and the end effector (spryTrack 180, Atracsys, Puidoux, Switzerland). The tissue-mimicking phantom consisted of an acrylamide polymer with a thermochromic ink (Kromagen MB 60-NH Magenta, LCRHallcrest, Flintshire, UK), whose property is to change color irreversibly from off-white to magenta when heated above 60 °C. This property served to mimic the coagulation effect of thermal ablation on human tissue when heated above 60 °C [21] and later was utilized for effective measurement of the ablation volume dimensions and shape.

### Calibration of ablation profiles

With the interaction between tissue and the energy emitted from the ablation device currently unknown [22], we propose to use a calibration technique to estimate the ablation width as a function of power and velocity, specific to the applied device and for each type of ablated tissue. To calibrate individual ablation systems and estimate the accuracy of predicted ablation shapes, a set of cylindrical ablation shapes were produced in a tissue-mimicking phantom, applying ablation powers between 60 and 120 W and velocities between 0.05 and 0.12 mm/s over a distance of 35 mm. We then applied the following probabilistic model:

$$\begin{array}{c}\beta=0\\\beta_{\mathrm{power}},\beta_{\mathrm{velocity}}\sim Normal\\\begin{array}{c}\varepsilon,\varepsilon_{\mathrm{power}},\varepsilon_{\mathrm{velocity}}\sim Exponential\\\mu_{\mathrm{width}}=\beta+\beta_{\mathrm{power}}\cdot\log\left(\mathrm{power}\right)+\beta_{\mathrm{velocity}}\cdot\log\left(velocity\right)\\\begin{array}{c}\sigma_{\mathrm{width}}=\varepsilon+\varepsilon_{\mathrm{power}}\cdot\mathrm{power}+\varepsilon_{\mathrm{velocity}}\cdot velocity\\width\mathit\sim Normal\left(\mu_{\mathrm{width}},\sigma_{\mathrm{width}}\right)\end{array}\end{array}\end{array}$$

The coefficients for the average predicted width ($\beta ,{\beta }_{\mathrm{power} },{\beta }_{\mathrm{velocity}}$) and for the uncertainty ($\varepsilon , {\varepsilon }_{\mathrm{power}},{\varepsilon }_{\mathrm{velocity}}$) are estimated using a Markov chain Monte Carlo sampler using the PyMC software package [23]. The code is available on Zenodo (https://doi.org/10.5281/zenodo.7542369) and technical details for the calibration are available in Additional file 1: Appendix 1.

### Proof-of-principle in clinical case ablation shapes

To proof the concept of the proposed dynamic energy delivery system and investigate its *ex vivo* applicability, four clinical cases of malignant liver tumors with typical tumor shapes smaller than 5 cm were selected from two public datasets (LiTS [24], 3D-IRCADb [25]).

**Fig. 6:**
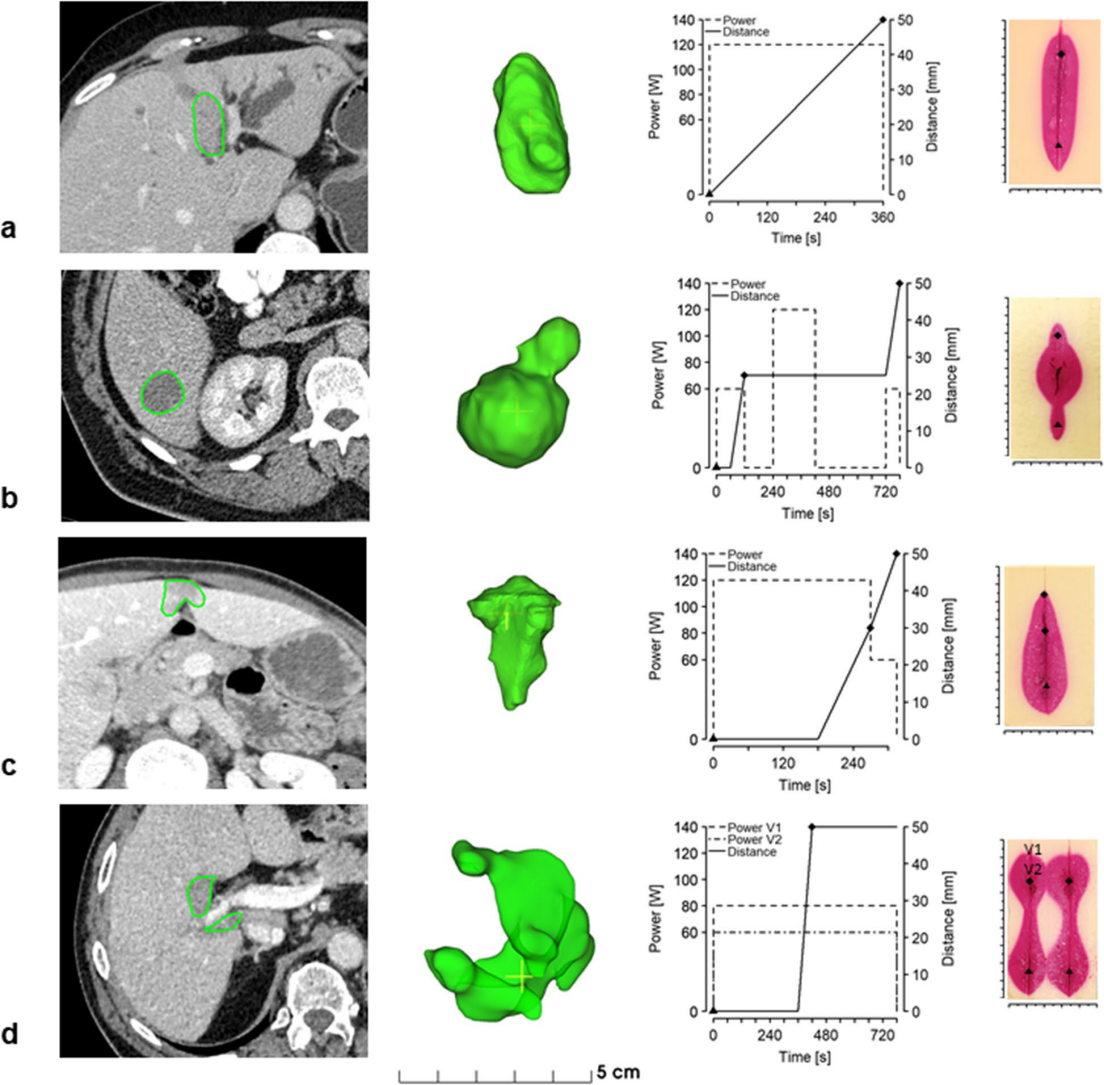
Left: axial view of computed tomography images with three-dimensional views of segmented tumors (green). Middle: corresponding ablation profiles, from the starting point (triangle symbol) to the ending/changing points (diamond symbols). **a** Constant velocity and constant power. **b** Stationary ablation followed by constant velocity and power. The pauses in power delivery between the segments ensured clear-cut shapes, stopping the heat from over-propagating as it occurs with continuous energy delivery. **c** Combination of stationary ablation followed by an interval of needle motion with decreasing power. **d** Multitrajectory ablation by sequencing two stationary ablations connected with a transient period, with constant power and second trajectory with a decrease by 20 W since specimen already heated from the first ablation. Right: resulting ablation shapes in the polymer phantom

Four ablation profiles corresponding to the selected ablation shapes were drafted (Fig. 6). A profile consisted of several discrete distance points from the ablation probe starting position, relative time frames, and respective ablation power values. These profiles were created using a custom software program built for the experiment execution. Data acquired from previously conducted calibration experiments were used to estimate the ablation power and ablation probe velocity settings required to achieve a certain ablation width at a given location. The ablation profile was sent to the robot via a software package, which translated it into straight-line trajectories with given velocities. Since available ablation systems do not offer a software API to integrate the robotic control software to date, the robot and the ablation device were controlled separately, using the timer on the ablation device to manually adjust the power setting on the ablation device according to the energy profile. After the creation of corresponding ablation volumes, the ablated specimens were cut along the ablation probe axis using a custom-made cutting tool. The cut specimens were then placed on a canvas with ArUco markers [26] and photographed. Using these markers, the pixel spacing was calculated, and the shape of the ablation volume was extracted using a color-based segmentation method.

## Results

**Fig. 7:**
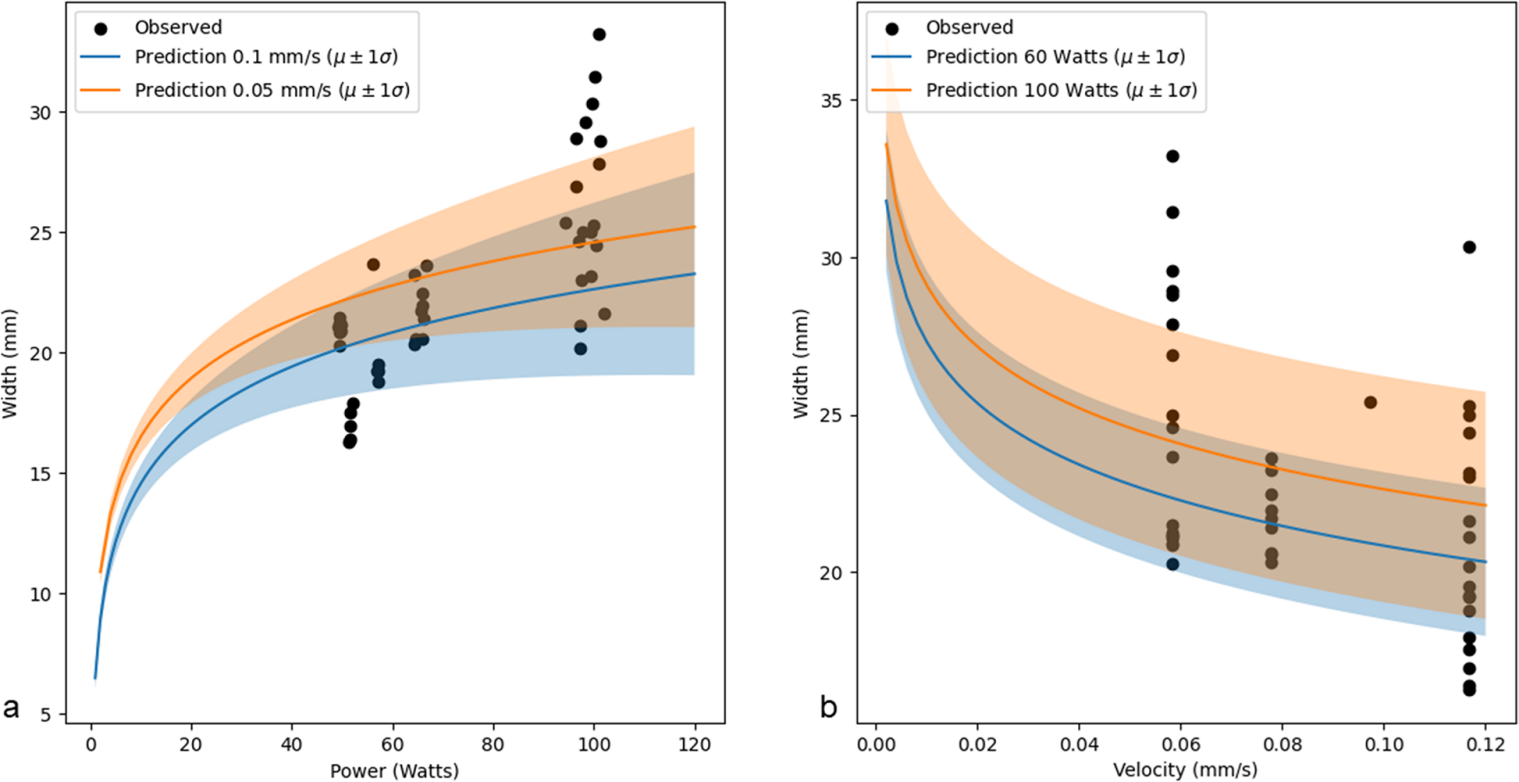
Predicted ablation widths with standard deviations (shadowed areas) as a function of power (**a**) and velocity (**b**)

Figure 7 shows the predicted widths depending on functions of power and velocity, confirming the logarithmic relationship between ablation width and power and velocity, within the applied ranges. The uncertainty (standard deviation) of the ablation width increases with increasing power by ± 0.03 mm (95% credible interval 95% 0.02–0.043) per watt increase in power and by ± 0.85 mm (95% credible interval 0–2.5) per mm/s increase in velocity. For other ablation modalities and devices, the relationship and the coefficients are likely different, and the calibration step should be performed for each device that will be used.

Using the calibrated experimental setup, corresponding ablation shapes were reproduced in the tissue-mimicking phantom. Graphical results of the selected clinical cases, planned ablation profiles, and resulting ablations are shown in Fig. 6. The main findings were that using faster movements and lower power resulted in narrower ablations and slower movements or higher powers resulted in wider ablations. These patterns could be combined to achieve wider and narrower areas along the ablation trajectory. Detailed measurements of the ablation shapes are presented in Table 1.

**Table 1:** Ablation shapes and size created to be custom to preselected clinical cases

Number	Tumor shape	Target ablation shape	Clinical challenge	Size of created ablation volumes			
				**Length (mm)**		**Width (mm)**	
**a**	Elongated	Cylindrical	Proximity to portal structures	75		20	
**b**	Dumbbell	Connected ellipsoids	Two adjacent tumor nodules	63		*p1*	8
						*p2*	26
						*p3*	9
**c**	Flattened	Teardrop	Subcapsular location	68		*p1*	25
						*p2*	17
**d**	Irregular	Connected spheres	Embracing portal vein	*Left*	91	*p1*	22
						*p2*	22
				*Right*	92	*p1*	24
						*p2*	24

*p* = marked (diamonds) positions along the ablation trajectory in Fig. 6

## Discussion

In this work, we introduced the concept of a fully automated treatment approach to bespoke thermal ablation, addressing the precision challenge in the ablation treatment of malignant tumors. The missing piece thus far is the creation of ablation shapes bespoke to the individual tumor volumetric appearance; we proposed a novel algorithm for dynamic energy delivery, combining microwave ablation technology with robotic guidance. We confirmed that bespoke ablation shapes can be created by simultaneous modulation of the ablation energy and ablation probe movement over time.

The clinical challenge of effective and safe thermal ablation lies in finding the balance between creating ablation volumes large enough to cover all tumor tissue and small enough to avoid injury to adjacent critical structures. Thus, obtaining a ratio of necessary to unnecessary ablated tissue close to one. This has previously been addressed by creating multiple overlapping ablation volumes, especially for the treatment of large and irregularly shaped tumors [27], *e.g.*, by simultaneously applying a multitude of ablation probes using stereotactic guidance. This requires extensive training and expertise and potentially compromises safety due to the creation of large areas of tissue necrosis and has therefore not gained broad application in the clinical community. We rather propose to aim for ablation volumes bespoke to individual tumor shapes in a precision medicine approach, which was used as a starting point to develop a novel algorithm for dynamic energy delivery.

We herein documented that with the proposed approach, complex ablation shapes can be created bespoke to individual tumor shapes by tuning the ablation power with reference to the probe position in our model (Table 1, Fig. 3). As anticipated based on the device specifications, resulting ablation volumes were rotationally symmetric around the probe axis and maintained rounded edges. The resulting two degrees of freedom of power and velocity were applied to modulate the width of the ablation shape and one degree of freedom to modulate the length (distance). This might vary with different ablation devices that would need to be calibrated accordingly. Other thermal ablation techniques such as laser ablation could potentially overcome the limitation of rotationally symmetric shapes and add a third degree of freedom for the ablation width (*i.e.*, rotation around the needle shaft) [28].

Overall, it was observed that (i) elongation of ablation volumes can be reached by continuous ablation during ablation probe retraction, (ii) radial shape distribution depends on the change in the delivered energy *versus* probe position (*e.g.*, round shapes by regular stationary ablation or pointy and narrow shapes by faster probe movements or pauses in energy delivery), and (iii) a higher variation in ablation size with higher power (see calibration paragraph), with high power should be used for larger uniform areas and lower power with low speeds for the boundary where higher accuracy is required. With sophisticated precision tools also with stereotactic and robotic guidance already available for the planning of ablation probe trajectories and highly precise ablation probe placement [15], we aimed to develop an automated solution for creating bespoke ablation volumes. In the proposed dynamic energy delivery algorithm, ablation probe movements with velocities below 1 mm/s are typically required for continuous ablation along a defined trajectory, which is not possible to deliver manually. Even though it can be assumed that the use of robotic arms will become standard in interventional radiology for general-purpose ablation and other interventions in the near future, currently available robotic systems like MAXIO (Perfint Healthcare, Chennai, India), Micromate (iSYS Medizintechnik, Untermeitingen, Austria), and ACE (XACT Robotics, Caesarea, Israel) [15] do not yet have the required dexterity for such slow movements. However, we can build upon the clinical setup used for stereotactic and robotic guidance with patient immobilization using vacuum mattresses, and breathing motion reduction using high-frequency jet ventilation to mitigate issues arising from organ motion and deformation [10]. Therefore, we are currently developing a robotic device as an extension of a stereotactic guidance system that is able to place the ablation probe, subsequently pull it back, and simultaneously control the ablation energy with respect to the pre-planned profile.

The other next step toward implementation of the proposed method is to create an automated interface between the ablation device and robot allowing monitoring and controlling energy settings. A collaboration with ablation device manufacturers to this end will be crucial. This will also allow developments such as a potential incorporation of additional parameters such as direct tissue temperature measurements during ablation, allowing automated feedback loops for ongoing validation and fine-tuning of created ablation volumes. The almost invariably present fluctuating energy output from ablation devices, currently impairing the predictability of created ablation volumes, could further be addressed. From a radiomics point of view, current image resolution is limited typically to 1 mm^3^ voxels and thus cell volumes in the evaluation of ablation volumes. If higher image resolution becomes available in the future, this could display ablation energy effects on a single-cell level and further enhance precision in ablation profile planning. The phantom used in this study did not allow computed 3D scanning; hence, we could not quantitatively compare the obtained ablation volumes with the planned shapes in 3D. Toward a clinical application of the proposed concept of dynamic energy delivery, the next step forward is to reproduce the creation of bespoke ablation volumes in perfused liver models, mimicking human tissue properties which significantly influence energy expansion and resulting final ablation volumes [20, 29]. We currently plan on using normothermic artificially perfused *ex-vivo* animal livers, as applied for graft perfusion in liver transplantation [30].

The incorporation of this novel dynamic energy delivery system into a comprehensive automated treatment model of automated trajectory and ablation profile planning [14] and ablation probe insertion [15] will optimally complement precision-focused thermal ablation treatment, especially in complex organs such as the liver. Automated treatment validation by quantitative ablation margin assessment [9] will further be integrated into a closed-loop system, which continues ablation treatment until all tumor tissue is ablated with the desired safety margin. This would potentially ensure 100% treatment success even if bespoke ablation leads to remaining tissue after the first round of ablation, adding enhanced safety and efficacy to thermal ablation treatment. This would allow a standardized minimally invasive treatment approach with a personalized treatment design, leading to reproducible clinical outcomes, probably allowing a broader application of ablation treatment and pushing the boundaries of treatment eligibility in patients with malignant tumors who would traditionally not be enrolled in ablation treatment.

The additional steps in such a workflow and even the potentially added cost or perceived complexity would therefore most probably be outweighed by the clinical benefit of bespoke ablation when using such a dynamic energy delivery system. Clinical implementation and proofing enhanced clinical outcomes would represent the next step once a clinically applicable device is available, which we are currently focusing on. Nevertheless, in light of the current clinical need in ablation procedures for complex tumors, good acceptability in the clinical community can be presumed. Furthermore, this technique could be applied not only for liver tumor ablation, but also in other organs such as the kidney or the lung, as well as for other procedures in need of a robotic arm for general-purpose tool positioning.

In conclusion, we introduced a concept for automated treatment of large and/or irregularly shaped malignant tumors including a novel dynamic energy delivery system. We demonstrated that the shape of the ablation volumes can be modified by simultaneous modulation of ablation energy and needle position over time. This potentially leads to the achievement of highly bespoke ablation shapes with more predictable clinical outcomes for the ablation of complex tumors.

### Supplementary Information

**Additional file 1: Appendix 1.** Code and technical details of calibration of ablation profiles. **Fig. S1.** Calibration of ablation profiles. Predicted ablation widths with standard deviations (shadowed areas) as a function of power (left) and velocity (right).

## Data Availability

Data and code are available on Zenodo (https://doi.org/10.5281/zenodo.7542369).
