# Supplementary material for: Thermal ablation with configurable shapes: a comprehensive, automated model for bespoke tumor treatment
Source: Eur Radiol Exp. 2023 Nov 7;7:67. doi: 10.1186/s41747-023-00381-6 (PMC10628015; doi:10.1186/s41747-023-00381-6)
Supplement: Supplementary file 1 — Additional file 1: Appendix 1. Code and technical details of calibration of ablation profiles. Fig. S1. Calibration of ablation profiles. Predicted ablation widths with standard deviations (shadowed areas) as a function of power (left) and velocity (right). [file 41747_2023_381_MOESM1_ESM.docx]

# Thermal ablation with configurable shapes: a comprehensive, automated model for bespoke tumor treatment

**ELECTRONIC SUPPLEMENTARY MATERIAL**

# Appendix 1

## Code and technical details of calibration of ablation profiles

To calibrate individual ablation systems and estimate the accuracy of predicted ablation shapes, a set of cylindrical ablation shapes were produced in a tissue mimicking phantom, applying ablation powers between 60 W and 120 W and velocities between 0.05 mm/s and 0.12 mm/s over a distance of 35 mm. The coefficients $\alpha, \beta_{power}, \beta_{velocity}$ from equation (2) were estimated using the Markov Chain Monte Carlo method^35^*.* Supplementary Figure 1 shows the predicted widths depending as functions of power and velocity, confirming the logarithmic relationship between ablation width and power and velocity, within the applied ranges. The uncertainty (standard deviation) of the ablation width increases with increasing power by ±0.03 mm (CI_95%_ [0.02, 0.043]) per Watt increase in power and by ±0.85 mm (CI_95%_ [0, 2.5]) per mm/s increase in velocity. For other ablation modalities and devices, the relationship and the coefficients are likely different, and the calibration step should be performed for each device that will be used.


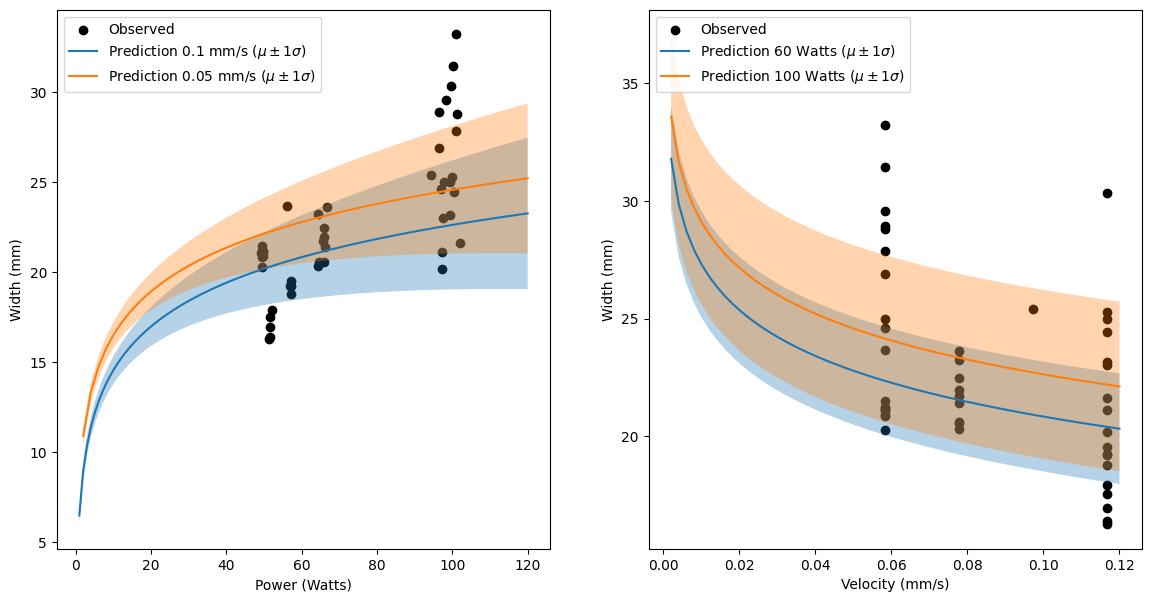


**Supplementary Figure 1 | Calibration of ablation profiles.** Predicted ablation widths with standard deviations (shadowed areas) as a function of power (left) and velocity (right)

*Code available in the code supplement.*
